# The prooxidant–antioxidant balance in diagnosis and developmental prognosis of premature neonates with asphyxia

**DOI:** 10.2478/abm-2024-0017

**Published:** 2024-06-28

**Authors:** Maryam Zakerihamidi, Boskabadi Hassan, Amirkhani Samin

**Affiliations:** Department of Midwifery, School of Medical Sciences, Tonekabon Branch, Islamic Azad University, Tonekabon 46617-34666, Iran; Department of Pediatrics, Faculty of Medicine, Mashhad University of Medical Sciences, Mashhad 91778-96149, Iran

**Keywords:** asphyxia, newborn, premature birth, prognoses, prooxidant antioxidant balance

## Abstract

**Background:**

The antioxidant system in a preterm neonate is premature. The imbalance between the prooxidant and antioxidant systems can make these neonates prone to oxidative stress. Birth asphyxia is one of the factors that can disturb this balance.

**Objective:**

We studied the prooxidant–antioxidant balance (PAB) in the diagnosis and developmental prognosis of preterm neonates with asphyxia.

**Methods:**

This cohort study has been conducted between 2016 and 2022 with 2 years follow-up on 183 premature neonates admitted to Ghaem Hospital Mashhad, by using a convenience sampling method. The data-collection tool and the researcher-made checklist included the mothers' and the neonate's information, and the third segment included laboratory information. PAB was studied by using standard solutions and the Enzyme immunoassays (ELISA) method. After discharging the newborns from the hospital, they were under follow-up at 6 months, 12 months, 18 months, and 24 months, by using the Denver II test. PAB was compared among newborns with asphyxia, those without asphyxia, and also newborns with normal and abnormal outcomes in both groups.

**Results:**

The mean ± standard deviation of the PAB factor reported is as follows: in newborns without asphyxia (21.00 ± 18.14 HK), those with asphyxia (31.00 ± 45.42 HK), in newborns with asphyxia having abnormal outcomes (40.00 ± 60.84 HK), and those having normal outcomes (21.00 ± 18.67 HK) (*P* ≤ 0.05). PAB results >25 HK have been used for the diagnosis of asphyxia prognosis in newborns, with 83.3% sensitivity and 81% specificity.

**Conclusion:**

The PAB index showed a significant increase after asphyxia. It can be used as a diagnostic marker for the prognosis of premature newborns with asphyxia. Thus, diagnosis and prognosis of asphyxia in premature newborns can be predicted by using the PAB index.

Preterm newborns are those that are born before 37 weeks of gestation. Despite the great progress made in the field of perinatology, the prevalence of preterm is increasing and it is considered an important factor associated with childhood complications [1]. Although the death rates of term and preterm neonates have significantly decreased over the past 3–4 decades, premature neonates are still prone to many diseases [2]. It is believed that about 23% of neonatal mortality in our Neonatal Intensive Care Unit (NICU) is related to birth asphyxia [3].

Severe prematurity is one of the most common reasons for neonatal mortality in our center [4].

The loss of blood circulation or the disruption in the gas exchange to the fetus is followed by prenatal asphyxia and can affect the neonate before, during, or after birth [5]. Prenatal asphyxia is one of the main reasons for brain damage and in severe cases, it can cause attention deficit hyperactivity disorder (ADHD), seizures, cerebral palsy (CP), multiple system dysfunction, and even death [6], [7], [8].

The factors related to the period before, during, or immediately after birth can play a major role in asphyxia occurrence [9]. Mother's ill health during pregnancy can lead to problems such as impaired fetal blood circulation, intrauterine growth retardation (IUGR), and difficulties during labor and neonatal asphyxia. Hence, there should be serious supervision immediately after birth. Asphyxia is a crucial and common issue in prenatal care [10]. The diagnosis of severe asphyxia is achieved by relying on the Apgar score, arterial blood gases, and hypoxic-ischemic encephalopathy (HIE) signs [10]. Many studies have been conducted toward studying the diagnostic biomarkers of asphyxia and its prognosis in term neonates. Many diagnostic biomarkers are used for the assessment of asphyxia; some of them are as follows: lactate, Lactate dehydrogenase (LDH), creatine kinase, adenylate kinase, prooxidant–antioxidant balance (PAB), interleukins S-100 β, neuron-specific enolase, brain-specific creatine kinase, neuro proteins, calcium bounded with proteins, vasoactive factors and inflammatory mediators [11], [12], [13], heat shock protein (HSP) [10, 14, 15], and also the number of nucleated red blood cells (NRBCs) found in umbilical cord blood [10], [11], [12], [14], [15], [16]. However, in preterm neonates, the diagnostic value of these markers still remains unknown.

There are various difficulties in diagnosing asphyxia in premature neonates. Many factors that are used for the indication of asphyxia in term neonates are not efficient in the diagnosis of asphyxia in preterm neonates. One of the differences between term and preterm neonates can be the cellular defense against asphyxia.

In preterm neonates, the defense systems are premature due to damages caused by asphyxia and ischemia, and may lead to multiple harmful consequences in these neonates. One of these premature systems is PAB. There is a delicate balance between the production and elimination of prooxidants in neonates. Oxidative stress is defined as the imbalance between prooxidants and antioxidants [9]. The increase in oxidants and decrease in antioxidants affects the pathogeneses of many diseases such as asphyxia. Following oxidative stress, cellular adaptive responses occur, which require certain processes for the production of antioxidants. As a result of oxidative stress, the deoxyribonucleic acid (DNA) of lipids and proteins is severely damaged. Oxidative stress is the result of an excessive increase in the production of oxidants or reactive types of oxygen [10].

Due to large intake of oxygen, the weakness of the antioxidant systems, its incapability of defending against hyperoxia, challenges early after birth, and the imbalance between the prooxidants and antioxidants systems, premature neonates are considerably prone to oxidative stress. The production of free radicals can result in oxidative damage to organs and systems throughout the body [17].

The imbalance between the prooxidants and antioxidant factors can increase the oxidative stress factors, which affect cell functions. Due to asphyxia and stress, the PAB changes in the body can turn to be an indicator of asphyxia [11]. The results of a study have shown that the mean serum PAB in term neonates having asphyxia increases [18]. In another study, PAB measurements and the degree of HIE altogether have been used for asphyxia diagnosis [19].

The role of oxidative stress in the pathogenesis and progress of prenatal asphyxia in preterm neonates is not quite known. One of the greatest shortcomings in this field can be the absence of an accurate and reliable method to measure the balance of the prooxidant–antioxidant simultaneously in these patients. A simple, fast, yet inexpensive method to measure PAB is by using 3,3,5,5 tetra methyl benzidine (TMB) cations. In addition, redox factor allows us to study PAB simultaneously [20].

The majority of studies in this field have been conducted on term neonates, and there is no enough information about preterm neonates at hand. The diagnosis of asphyxia can have an important role in accelerating asphyxia treatment in premature neonates. Considering the high prevalence of asphyxia, its long-term impacts on premature neonates, and the fact that there is no specific standard for the diagnosis and long-term prognosis of asphyxia, this study has been conducted for researching the diagnosis and prognosis of neonates with asphyxia by using the oxidative factor PAB.

## Methods

The approval to conduct this study was provided by the ethics committee of Mashhad University of Medical Sciences (IR. MUMS. REC.1394.57). The written informed consent was obtained from the guardians of the newborns.

In a prospective cohort study, we studied the PAB in preterm neonates with and without asphyxia whose blood sample had been taken 1 h after birth. Prognosis at 2-years-old was studied at Ghaem Hospital Mashhad, Iran, during the years 2016–2022, by convenience sampling method. Neonates with a gestational age <37 weeks with asphyxia were included in this study. Neonates with at least two of the below-mentioned symptoms or signs were considered to have asphyxia:

- History of detectable complications during labor (placental abruption, uterine rupture, umbilical cord prolapse) accompanied by fetal intrapartum monitoring disorders.
- Potential of Hydrogen Ion (pH) <7.2 in fetal blood sample/umbilical cord/first hour after birth.
- Multiple system disorders in <48 h after birth, such as kidney, liver, and heart dysfunctions.
- Excessive need for ventilation (>72 h) while not having respiratory diseases/neuromuscular diseases.
- Delaying in the improvement of metabolic acidosis (>24 h).
- Specific area damage (white matter injury, periventricular leukomalacia (PVL), basal ganglia without affecting the cerebral cortex) in Magnetic Resonance Imaging (MRI) examination.

Premature neonates born without the clinical signs of asphyxia mentioned above and without the need for resuscitation in the delivery room were included as the control group.

Exclusion criteria were: congenital defects, congenital infections, and chorioamnionitis. To evaluate PAB, two ccs of blood were taken from the umbilical cord while performing other tests.

The PAB was calculated by using TMB in two different reactions: (1) an enzymatic reaction in which chromogens were oxidized into cationic TMB by peroxide, which in this test is (H_2_O_2_) and (2) the other is a reaction in which cationic is revived by antioxidants (in this test, it is uric acid). For this purpose, 16 mg of TMB powder was dissolved into 10 mL of dimethyl sulfoxide (DMSO) until the TMB/DMSO solution was obtained. Afterward, to create a cationic TMB solution, 400 mL of the above solution was added to 20 mL of the sodium buffer acetate solution (pH = 4.5/0.05 M). About 70 mL of recently made chloramine-T (100 mM) was added to 20 mL of this solution, mixed well, and incubated for 2 h in a darkened room and at room temperature. In the second step, 25 U of the peroxidase enzyme solution was added to 20 mL of cationic TMB solution and 1 cc was distributed among microtubes and kept at 20°C temperature. To prepare the TMB solution, 200 mL of the Tetra Methyl Benzidine (TMB)/DMSO solution was added to 10 mL of sodium buffer acetate (pH = 5.8/0.05 M) and then 1 mL of cationic TMB solution, which had peroxidase enzyme (step two solution), would be mixed with 10 mL of the TMB solution and incubated for 2 min at room temperature in a darkened room. About 10 mL of each standard serum sample or blank (distilled water) with 200 mm of the (third step solution) has been mixed and injected into Elisa and was incubated for 12 min at 37° temperature.

From the information gained from the standard samples (uric acid and H_2_O_2_' 20%, 25%, 50%, 75%, 100%) with specified concentrations, a standard curve was obtained, which can be used to determine the concentration of the said samples at 450 nm wavelength. From the standard samples, a standard curve will be obtained that can demonstrate the PAB value by Hamidi-Koliakos (HK) unit. This curvature shows the effects of hydrogen peroxide on the standard solution. The value of the samples is calculated on a basic basis and their amount is shown on the curved lines [20].

Patient evaluation has been done based on clinical examinations, needed laboratory surveys, and in case of having scientific indications, by using imaging methods such as chest X-Ray, ultrasound, or MRI.

The control group included neonates admitted to the Neonatal Intensive Care Unit (NICU) who did not show any signs of asphyxia. The data related to this study have been categorized into 3 sections by using the researchers' checklist. The first and second sections included the data related to the mother (mother's age, parity, difficulties during labor, gestational age) and the neonates (gestational age, birth weight, first minute and fifth minute Apgar score), respectively, and the third segment included the laboratory information (PAB in umbilical cord blood).

Neonates were under follow-up after being discharged in 6 months, 12 months, 18 months, and 24 months by using the Denver II test. Denver II developmental screening test is an international test used for monitoring children's growth and evolution, from birth until the age of 6, which covers 4 functions: gross motor, language, fine motor-adaptive, and personal-social, and is widely used for diagnosis of speech and learning disorders, autism, mild to mid-mental retardation and psychosocial complications.

The items in Denver II have been carefully selected in terms of reliability and comprehensiveness of norms across all subgroups and cultures. If the infant had a problem in one of the 4 areas, it would be considered a developmental delay.

In case of having a problem in only one area, it would be considered as mild developmental delay, in two areas, moderate developmental delay, and in three or above areas, the neonate would be considered to have severe developmental delay [21].

Neonatal outcomes caused by asphyxia were investigated in both groups with normal and adverse outcomes. Adverse outcomes included developmental delay and death.

### Statistical calculation

The outcomes of neonates with asphyxia were analyzed after follow-up. In the case of being normal in all areas, it would be considered as a normal outcome, and in the case of having at least one problem in one area or death, it was considered as an abnormal outcome. First, by using figures and tables, the outcomes were described, and then chi-squared test and *t* test were used. Also, to indicate predicting factors in preterm neonates, regression models were used for indicating the diagnostic value of PAB. In the prognosis and diagnosis of asphyxia, the Receiver Operating Characteristic (ROC) curve was used. In all cases, *P* ≤ 0.05 was considered.

## Results

In the current study, 239 premature neonates were enrolled. In total, 150 neonates (66.7%) were without asphyxia and 89 newborns (33.3%) were diagnosed with asphyxia.

Twenty-eight neonates died during the first 2 months (9 in the group of neonates without asphyxia and 19 in the group of neonates with asphyxia).

**Figure 1.**
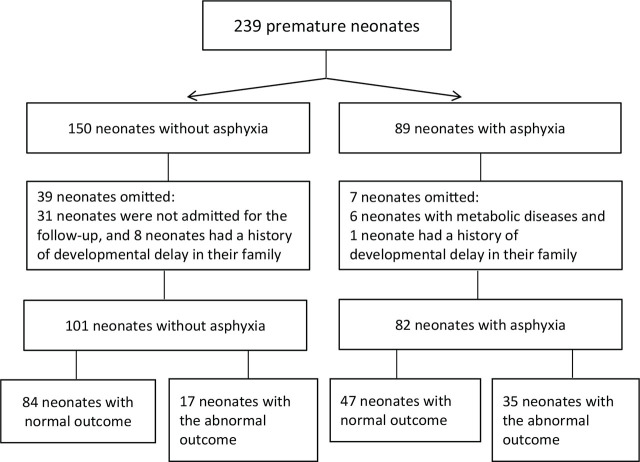
Selected neonates.

From the 239 premature neonates under study, we were able to follow up on 183 of the cases up to 2 years, and were not able to follow up on 46 cases (31 neonates were not admitted for the follow-up, 6 neonates were diagnosed with metabolic diseases, and 9 of them had a history of developmental delay in their family) (**Figure 1**).

The number of neonates admitted at the beginning of the study was 229, 186 at 6 months, 173 at 1 year, 164 at 18 months, and 155 at 2 years follow-up.

In neonates with asphyxia (82 neonates), 47 neonates (57.3%) had normal outcomes and 35 neonates (42.7%) had abnormal outcomes. Of the neonates without asphyxia (101 neonates), 84 of them (75.24%) had normal outcomes and 17 neonates (14.86%) had abnormal outcomes.

For neonates studied in our research, the mean and standard deviation gestational age was 32.31 ± 2.70 weeks, birth weight was 1647.46 ± 614.75 g, and PAB was 31.62 ± 24.91 HK.

**Figure 2.**
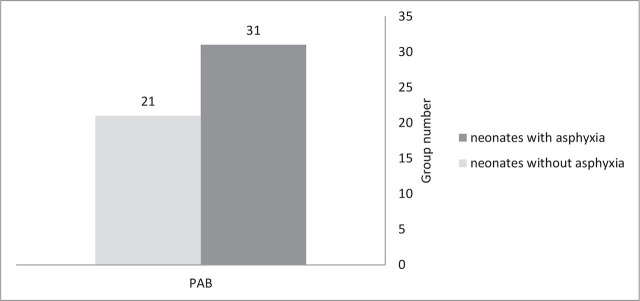
The comparison of mean PAB in neonates with and without asphyxia. PAB, prooxidant–antioxidant balance.

**Figure 3.**
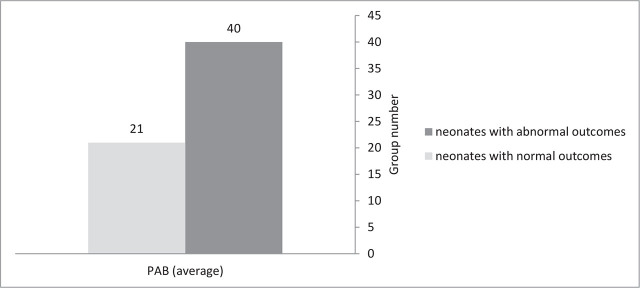
The comparison of PAB in the two groups of neonates with normal or abnormal outcomes. PAB, prooxidant–antioxidant balance.

The two groups of neonates without and with asphyxia had a significant statistical difference in mother's age, gestational age, birth weight, first pH, and first- and fifth-minute Apgar Score **(Table 1)**. The value of serum PAB in neonates with asphyxia was reasonably more (*P* = 0.019) (**Figure 2**). The t-test outcomes show that PAB (*P* = 0.000), in the two groups of neonates with normal outcomes and abnormal outcomes had a significant difference (**Figure 3**). The value of PAB was higher in neonates with abnormal outcomes.

**Table 1.** The comparison between clinical and laboratory parameters of mothers and neonates in two groups of neonates with asphyxia and without asphyxia

**Variables Groups**	**Neonates without asphyxia *n* = 82**	**Neonates with asphyxia *n* = 101**	***P* (T–Test)**
Mother's age (year)	7.99 ± 27.12	6.50 ± 29.69	0.006
Parity	1.48 ± 2.02	1.39 ± 2.18	0.368
Gestational age (week)	32.45 ± 2.48	33.55 ± 3.00	0.013
First PH	7.29 ± 0.10	7.19 ± 0.13	0.000
First minute Apgar Score	7.20 ± 1.69	4.32 ± 1.49	0.000
Fifth minute Apgar score	8.70 ± 1.26	6.47 ± 1.81	0.000
Birth Weight (gr)	1575.24 ± 505.61	2135.89 ± 749.48	0.000

The values are based on standard deviation ± mean.

In the current study, the PAB above the cutoff 25 HK, in the two groups of neonates with normal outcomes and abnormal outcomes, had a meaningful difference (*P* = 0.000). Taking the ROC line into consideration, the PAB sensitivity for the diagnosis of asphyxia prognosis in preterm neonates was 83.3% and its specificity was 81%.

In terms of asphyxia severity, 32 neonates did not have HIE, 30 neonates had Hypoxic ischemic encephalopathy (HIE)1, 10 neonates had HIE2, and 10 neonates had HIE3. The PAB in the group of neonates without HIE was 17.17 (13.22), with HIE1 (12.40) 21.12, HIE2 (19.37) 27.00, and with HIE3 was (32.54) 47.07.

Normal and adverse neonatal outcomes (developmental delay and death) in the group of neonates with asphyxia and the group of neonates without asphyxia were investigated.

**Figure 4.**
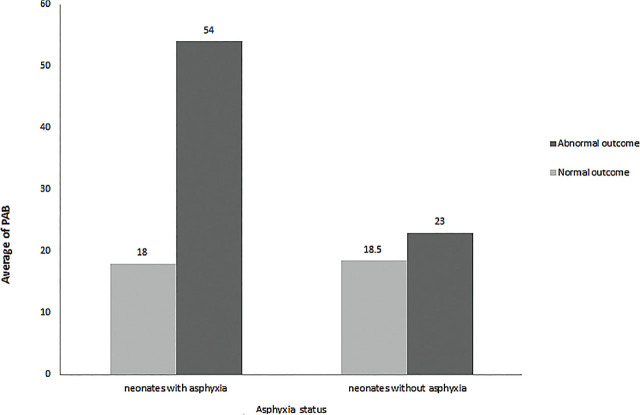
The comparison of PAB in two groups of neonates based of having or not having asphyxia; having normal or abnormal outcomes. PAB, prooxidant–antioxidant balance.

In this study, the PAB variable in the group of neonates with asphyxia having normal and abnormal outcomes (*P* = 0.002) had a significant data difference. The incidence of this variable in neonates with asphyxia and abnormal outcomes was three times higher than in neonates with asphyxia and normal outcomes. Also, the PAB variable did not have a significant difference between the group of neonates without asphyxia with normal and abnormal outcomes (*P* = 0.253) (**Figure 4**).

Furthermore, in this study, the severity of asphyxia is different between groups, and the clinical severity or PAB correlated better to clinical outcomes.

## Discussion

According to our research, the value of PAB in neonates with asphyxia was about 1.5 times more than in neonates without asphyxia. In one study, the mean serum PAB in neonates with asphyxia was two times more than in healthy neonates. In a study by Boskabadi et al. [11], the PAB level in neonates with asphyxia was three times more than in healthy term neonates. Results of Aydemir et al.'s [22] study show that the PAB in the cases of prenatal asphyxia is disturbed and is in favor of prooxidants, and the degree of oxidative stress is related to the severity of the nervous system involvement in the neonates' first days of life.

The outcomes of Bahbah et al.'s [23] study depict that the capacity of combating oxidative stress in premature neonates compared to healthy term neonates was reduced. Prenatal asphyxia was considered one of the main reasons for the occurrence of oxidative stress during the infancy period.

Premature neonates are born before the formation of antioxidant systems. These antioxidant systems are capable of neutralizing the free oxygen radicals. Birth by itself can be a reason for oxidative stress, which when combines with other agents, namely, hypoxia, asphyxia, hyperoxia, reperfusion, or inflammation, can disturb the preterm neonates' defense mechanisms [24]. Therefore, analyzing the levels of serum PAB can be beneficial for the early diagnosis of prenatal asphyxia. Preterm newborns are prone to oxidative damage due to a reduction in the antioxidant enzyme activities such as catalase and glutathione peroxidase. Also, the imbalance between prooxidants and antioxidants can cause oxidative damage [25].

In the current study, >42.7% of the newborns with asphyxia had abnormal outcomes. In a study on term neonates, 23.5% of the neonates with asphyxia had abnormal prognoses [26].

In a study conducted on term neonates, the incidence of PAB in HIE grades one, two, and three was 7.03 HK, 19.4 HK, and 21.6 HK, respectively [27]. It has been indicated that fetal and neonatal antioxidant systems are premature and therefore they are prone to harmful effects of oxidative stress. Free radicals are highly reactive substances that can cause cell death or apoptosis with Self-reinforcing chain reactions. Oxidative stress damage can occur when the balance between the antioxidant enzymes produced and the free radicals changes in favor of free radicals. Therefore, oxidative stress plays a role in many fetal and neonatal diseases, which are usually caused by hypoxia [28].

According to the findings of our study, the incidence of PAB in the umbilical cord of premature neonates with asphyxia and having abnormal development was about three times more than in neonates with asphyxia having normal development (21.00 ± 18.67 HK compared to 40.00 ± 60.84 HK). While in another study, the mean serum PAB in term neonates with asphyxia having normal outcomes and abnormal outcomes was 17/1 ± 9/23 HK and 48/27 ± 41/30 HK, respectively [21].

Oxygen free radicals are responsible for brain damage following the occurrence of neonatal asphyxia. In the first stage of asphyxia reoxygenation, changes are made in the antioxidant enzyme activity, which can have an essential role in turning on and off the cascade reactions that damage neurons.

Hypoxia/ischemia causes the brain to activate its inner mechanisms such as antioxidant enzymes to set right the disturbed or lost neural circuits [29]. During hypoxic-ischemic cycles and reoxygenation, the inner production of superoxide radicals increases following the reduction of nicotinamide adenine dinucleotide phosphate (NADPH) [30]. In normal physiological conditions, antioxidant enzymes can offer protection against cell damage. However, during asphyxia, the antioxidant barrier would be inadequate for protection. Also, disturbances in PAB can occur following neonatal asphyxia impacting oxidative processes, which can lead to oxidative stress and hence neurological damage [31]. The brain is prone to oxidative stress because of the following reasons: neurons' membranes are filled with polyunsaturated fatty acids which are an important source of oxygen free radicals; antioxidant enzymes activity noticeably reduce in the brain; some areas of the brain are rich in iron and when the iron is not attached to protein, it has a prooxidative effect that can lead to the production of free radicals with harmful effects [32].

In the current study, the PAB >25 HK in the group of neonates with abnormal outcomes was 7 times more than in the group of neonates with normal outcomes. In this study, the PAB value of >25 HK was used for diagnosis of asphyxia prognosis in premature neonates; its sensitivity was 83.3% and its specificity was 81%. Outcomes of a study have shown that PAB >11.3 HK was used for diagnosis of asphyxia prognosis in premature neonates, and its sensitivity was 84% and the specificity was 92% [27]. In another study, combining the HIE intensity and PAB was used for predicting the outcomes in neonates with asphyxia, which had a high diagnostic value of 95.2% [21].

The main limitations of this study are considered to be our control group, which included hospitalized newborns that did not show any signs of asphyxia. Additionally, we were not able to follow up on the significant number of neonates admitted under the study.

Considering that the PAB index in asphyxiated neonates, as well as in asphyxiated neonates with abnormal outcomes, it can be used as an accurate test with high sensitivity and specificity to evaluate the prognosis of asphyxiated neonates.

## References

[1] Quinn J-A, Munoz FM, Gonik B, Frau L, Cutland C, Mallett-Moore T (2016). Preterm birth: case definition and guidelines for data collection, analysis, and presentation of immunisation safety data. Vaccine.

[2] Behrman RE, Butler AS, Institute of Medicine (US) Committee on Understanding Premature Birth and Assuring Healthy Outcomes (2007). Preterm Birth: Causes, Consequences, and Prevention.

[3] Liu L, Oza S, Hogan D, Chu Y, Perin J, Zhu J (2016). Global, regional, and national causes of under-5 mortality in 2000–15: an updated systematic analysis with implications for the Sustainable Development Goals. Lancet.

[4] Boskabadi H, Parvini Z, Barati T, Moudi A (2012). Study of the causes and predisposing factors in neonatal mortality in Ghaem Hospital (March 2009 To May 2010). Iran J Obstetr Gynecol Infertil.

[5] Goldenberg RL, Harrison MS, McClure EM (2016). Stillbirths: the hidden birth asphyxia—US and Global perspectives. Clin Perinatol.

[6] Usman F, Imam A, Farouk ZL, Dayyabu AL (2019). Newborn mortality in Sub-Saharan Africa: why is perinatal asphyxia still a major cause?. Ann Glob Health..

[7] Wosenu L, Worku AG, Teshome DF, Gelagay AA (2018). Determinants of birth asphyxia among live birth newborns in University of Gondar referral hospital, northwest Ethiopia: a case-control study. PLoS One.

[8] Peeva V, Golubnitschaja O (2009). Birth asphyxia as the most frequent perinatal complication. Predictive diagnostics and personalized treatment: dream or reality. Nova Science Pub Inc.

[9] Aslam HM, Saleem S, Afzal R, Iqbal U, Saleem SM, Shaikh MW, Shahid N (2014). “Risk factors of birth asphyxia”. Ital J Pediatr.

[10] Boskabadi H, Afshari JT, Ghayour-Mobarhan M, Maamouri G, Shakeri M, Sahebkar A, Ferns G (2010). Association between serum interleukin-6 levels and severity of perinatal asphyxia. Asian Biomed (Res Rev News).

[11] Boskabadi H, Omidian M, Tavallai S, Mohammadi S, Parizadeh M, Ghayour Mobarhan M, Ferns GA (2015). Serum Hsp70 antigen: early diagnosis marker in perinatal asphyxia. Iran J Pediatr.

[12] Naithani M, Simalti AK (2011). Biochemical markers in perinatal asphyxia. J Nepal Paediatr Soc.

[13] Bersani I, Auriti C, Ronchetti MP, Prencipe G, Gazzolo D, Dotta A (2015). Use of early biomarkers in neonatal brain damage and sepsis: state of the art and future perspectives. Bio Med Res Int.

[14] Volpe JJ (2001). Perinatal brain injury: from pathogenesis to neuroprotection. Ment Retard Dev Disabil Res Rev.

[15] Low JA, Pickersgill H, Killen H, Derrick EJ (2001). The prediction and prevention of intrapartum fetal asphyxia in term pregnancies. Am J Obstet Gynecol.

[16] Boskabadi H, Maamouri G, Sadeghian MH, Ghayour-Mobarhan M, Heidarzade M, Shakeri MT, Ferns G (2010). Early diagnosis of perinatal asphyxia by nucleated red blood cell count: a case-control study. Arch Iran Med.

[17] Lembo C, Buonocore G, Perrone S (2021). Oxidative stress in preterm newborns. Antioxidants (Basel).

[18] Boskabadi H, Navaee Boroujeni A, Mostafavi-Toroghi H, Hosseini G, Ghayour-Mobarhan M, Hamidi Alamdari D (2014). Prooxidant-antioxidant balance in perinatal asphyxia. Indian J Pediatr.

[19] Bassani DG, Kumar R, Awasthi S, Morris SK, Paul VK, Shet A, Million Death Study Collaborators (2010). Causes of neonatal and child mortality in India: a nationally representative mortality survey. Lancet.

[20] Alamdari DH, Paletas K, Pegiou T, Sarigianni M, Befani C, Koliakos G (2007). A novel assay for the evaluation of the prooxidant–antioxidant balance, before and after antioxidant vitamin administration in type II diabetes patients. Clin Biochem.

[21] Boskabadi H, Zakerihamidi M, Heidarzadeh M, Avan A, Ghayour-Mobarhan M, Ferns GA (2017). The value of serum pro-oxidant/antioxidant balance in the assessment of asphyxia in term neonates. J Matern Fetal Neonatal Med.

[22] Aydemir O, Akar M, Uras N, Eras Z, Erdeve O, Oguz SS, Dilmen U (2011). Total antioxidant capacity and total oxidant status in perinatal asphyxia in relation to neurological outcome. Neuropediatrics.

[23] Bahbah MH, Deeb MM, Ragab SM, El-Shafie MA (2015). Study of oxidative stress in common neonatal disorders and evaluation of antioxidant strategies. Menoufia Med J.

[24] Sahni PV, Zhang J, Sosunov S, Galkin A, Niatsetskaya Z, Starkov A (2018). Krebs cycle metabolites and preferential succinate oxidation following neonatal hypoxic-ischemic brain injury in mice. Pediatr Res.

[25] Marseglia L, D'Angelo G, Manti S, Arrigo T, Barberi I, Reiter RJ, Gitto E (2014). Oxidative stress-mediated aging during the fetal and perinatal periods. Oxid Med Cell Longev.

[26] Boskabadi H, Ashrafzadeh F, Doosti H, Zakerihamidi M (2015). Assessment of risk factors and prognosis in asphyxiated infants. Iran J Pediatr.

[27] Boskabadi H, Maamouri G, Ghayour-Mobarhan M, Bagheri F, Zakerihamidi M, Mollaey MK (2017). Comparison of the predictive value of prooxidant-antioxidant balance and heat shock proteins in the diagnosis of neonatal asphyxia. Biomed Res Ther.

[28] Perrone S, Santacroce A, Longini M, Proietti F, Bazzini F, Buonocore G (2018). The free radical diseases of prematurity: from cellular mechanisms to bedside. Oxid Med Cell Longev.

[29] Frankenburg WK, Dodds J, Archer P, Shapiro H, Bresnick B (1992). The Denver II: a major revision and restandardization of the Denver Developmental Screening Test. Pediatrics.

[30] Tarafdar A, Pula G (2018). The role of NADPH oxidases and oxidative stress in neurodegenerative disorders. Int J Mole Sci.

[31] Tokarz P, Kaarniranta K, Blasiak J (2013). Role of antioxidant enzymes and small molecular weight antioxidants in the pathogenesis of age-related macular degeneration (AMD). Biogerontology.

[32] Sola A, Rogido MR, Deulofeut R (2007). Oxygen as a neonatal health hazard: call for detente in clinical practice. Acta Paediatr.

